# Ginsenoside Rb1 ameliorates CKD‐associated vascular calcification by inhibiting the Wnt/β‐catenin pathway

**DOI:** 10.1111/jcmm.14611

**Published:** 2019-08-19

**Authors:** Peng Zhou, Xinyu Zhang, Mengqi Guo, Rong Guo, Lei Wang, Zihao Zhang, Zongwei Lin, Mei Dong, Hongyan Dai, Xiaoping Ji, Huixia Lu

**Affiliations:** ^1^ The Key Laboratory of Cardiovascular Remodeling and Function Research, Chinese Ministry of Education, Chinese National Health Commission and Chinese Academy of Medical Sciences, The State and Shandong Province Joint Key Laboratory of Translational Cardiovascular Medicine, Department of Cardiology Qilu Hospital of Shandong University Jinan China; ^2^ Department of Cardiology Ji’an Municipal Center People's Hospital Ji’an China; ^3^ Department of Cardiology Qingdao Municipal Hospital Qingdao China

**Keywords:** ginsenoside Rb1, PPAR‐γ, vascular calcification, Wnt/β‐catenin

## Abstract

Vascular calcification (VC) is a pathological process underpinning major cardiovascular conditions and has attracted public attention due to its high morbidity and mortality. Chronic kidney disease (CKD) is a common disease related to VC. Ginsenoside Rb1 (Rb1) has been reported to protect the cardiovascular system against vascular diseases, yet its role in VC and the underlying mechanisms remain unclear. In this study, we established a CKD‐associated VC rat model and a β‐glycerophosphate (β‐GP)‐induced vascular smooth muscle cell (VSMC) calcification model to investigate the effects of Rb1 on VC. Our results demonstrated that Rb1 ameliorated calcium deposition and VSMC osteogenic transdifferentiation both in vivo and in vitro. Rb1 treatment inhibited the Wnt/β‐catenin pathway by activating peroxisome proliferator‐activated receptor‐γ (PPAR‐γ), and confocal microscopy was used to show that Rb1 inhibited β‐catenin nuclear translocation in VSMCs. Furthermore, SKL2001, an agonist of the Wnt/β‐catenin pathway, compromised the vascular protective effect of Rb1. GW9662, a PPAR‐γ antagonist, reversed Rb1's inhibitory effect on β‐catenin. These results indicate that Rb1 exerted anticalcific properties through PPAR‐γ/Wnt/β‐catenin axis, which provides new insights into the potential theraputics of VC.

## 1 INTRODUCTION

Vascular calcification (VC) is a strong prognostic marker of cardiovascular disease mortality, and VC can be classified into intima‐based VC and media‐based VC.^1^ Chronic kidney disease (CKD), diabetes mellitus and ageing always complicate medial VC. In addition to kidney damage, vascular stiffness by CKD‐associated VC also leads to escalated cardiovascular mortality.^2^ Though passive deposition of hydroxyapatites is considered the main pathological process of VC, recent studies have reported VC to be a complex process with involvement of different molecular pathways, which includes active transformation of vascular smooth muscle cell (VSMC) into osteoblast‐like cells.^3^

The Wnt/β‐catenin molecular pathway was identified as a growth control pathway involved in differentiation processes, such as deciding the fate of stem cells.^4^ The degradation of cytoplasmic β‐catenin is inhibited when the pathway is activated, and subsequently, accumulated β‐catenin enters the nucleus where it regulates downstream gene expression.^5^, ^6^ Increasing evidence has indicated that the activation of the Wnt/β‐catenin pathway plays a key role in VC.^7^, ^8^, ^9^ For example, the Wnt/β‐catenin pathway promoted VSMC osteogenic transdifferentiation by modulating RUNX2 expression.^7^ Furthermore, inhibition of the Wnt/β‐catenin pathway was demonstrated to ameliorate VC.^10^

Peroxisome proliferator‐activated receptor gamma (PPAR‐γ) belongs to the nuclear hormone receptor superfamily. PPAR‐γ is expressed in various cell types and regulates certain cellular metabolism and differentiation processes.^11^ The mutual antagonism between PPAR‐γ and the Wnt/β‐catenin pathway has been demonstrated to regulate the differentiation of mesenchymal stem cells (MSCs) into adipocytes and osteoblasts.^12^ In addition, pioglitazone, a PPAR‐γ agonist, has been suggested to inhibit the canonical Wnt pathway, which attenuates VC.^13^

Ginsenosides are major components of the traditional herbal medicine ginseng, and ginsenosides can be classified into panaxadiols (PPD) and panaxatriols (PPT) by chemical structure. Rb1, the most abundant constituent of panaxadiol, has recently been reported to exert protective properties in vascular diseases and kidney diseases.^14^, ^15^, ^16^, ^17^ A clinical study indicated that Rb1 alleviated creatine and inflammatory cytokine levels in CKD patients.^18^ Furthermore, it has been reported that Rb1 reduced type I collagen expression through PPAR‐δ, which is considered an osteogenic profile marker of VSMC.^19^, ^20^ However, the role of ginsenoside Rb1 in CKD‐associated VC has not yet been studied.

In view of the demonstration that Rb1 acts as a PPAR‐γ agonist in human umbilical vein endothelial cells (HUVECs) by our previous study,^21^ we hypothesized that ginsenoside Rb1 may protect VC through the PPAR‐γ/β‐catenin axis. To test our hypothesis, we established a CKD rat model and a VSMC calcification model to mimic clinical CKD‐associated VC in vivo and in vitro.

## 2 MATERIALS AND METHODS

### 2.1 Reagents and antibodies

Foetal bovine serum (FBS) and Dulbecco's Modified Eagle's medium (DMEM) were obtained from Gibco‐BRL (Thermo Fisher Scientific, Inc). β‐Glycerophosphate (β‐GP; G5422) and adenine (V900471) were purchased from Sigma‐Aldrich (Merck KGaA). Ginsenoside Rb1 (41753‐43‐9) was obtained from Fleton Natural Products Co., Ltd. The Wnt/β‐catenin agonist SKL2001 (S8320) and PPAR‐γ inhibitor GW9662 (S2915) were from Selleck Chemicals. The Nuclear and Cytoplasmic Protein Extraction Kit (P0027) was from Beyotime Biotechnology.

Antibodies against calponin 1 (#17819), runt‐related transcription factor 2 (RUNX2; #12556), β‐catenin (#8480), phospho‐β‐catenin (Ser675) (#9567), glycogen synthase kinase‐3β (GSK‐3β; #9315), phospho‐GSK‐3β (Ser9) (#9322) and histone‐H3 (#4499) were from Cell Signaling Technology, Inc. An antibody against α‐smooth muscle actin (α‐SMA; NBP2‐22120) was purchased from Novus Biologicals. An antibody against peroxisome proliferator‐activated receptor gamma (PPAR‐γ; sc‐7273) was purchased from Santa Cruz Biotechnology. An antibody against glyceraldehyde‐3‐phosphate dehydrogenase (GAPDH; TA‐08) was purchased from ZSGB‐BIO.

### 2.2 Cell culture

Primary rat VSMCs were extracted from 8‐week‐old non‐CKD male Wistar rats. Briefly, the rats were killed by sodium pentobarbital, and the thoracic aorta was dissected out quickly. After removing the adventitia and intima, the artery segment was cut into 1‐2 mm^2^ sections and placed in a cell culture flask with DMEM containing 4.5 g/L glucose supplemented with 20% FBS, 10 mmol/L sodium pyruvate, 100 U/mL penicillin and 100 μg/mL streptomycin (growing medium). Cells were incubated at 37°C in a humidified atmosphere containing 5% CO_2_. Immunocytochemical examination showed positive staining in all cells for α‐smooth muscle actin. Migrated VSMC passages 3‐8 were used for in vitro experiments.

Vascular smooth muscle cells were seeded onto a 6‐ or 12‐well dish and incubated with DMEM + 10% FBS (Gibco; Thermo Fisher Scientific, Inc), and 10 mmol/L β‐GP (Sigma‐Aldrich) was introduced to induce VSMC calcification for 3‐12 days. The culture medium was replaced every 3 days. For drug‐treated groups, VSMCs were preincubated with different concentrations of Rb1 for 1 hour, followed by β‐GP calcification induction together with Rb1. Furthermore, SKL2001, an agonist of the Wnt/β‐catenin pathway as reported,^22^, ^23^ was applied to study pathway alteration. VSMCs were pre‐treated with 5 μmol/L GW9662 or different doses of SKL2001 (Selleck Chemicals) for 30 minutes, followed by 40 μmol/L Rb1 treatment with or without 10 mmol/L β‐GP.

### 2.3 Animal experiments

All animal experimental protocols complied with the Animal Management Rules of the Chinese Ministry of Health (document no. 55, 2001) and conformed to the NIH guidelines (the Guide for the Care and Use of Laboratory Animals published by the National Institutes of Health; NIH Publication No. 85‐23, revised 1996).

Male Wistar rats (160‐180 g) were randomly divided into three groups: control, CKD and CKD with ginsenoside Rb1 (CKD + Rb1), n = 5 per group. For CKD and CKD + Rb1 rats, adenine was administered intragastrically at a dose of 250 mg/kg/d for 2 weeks, followed by 250 mg/kg every other day for 4 weeks to establish CKD models based on the previous report.^24^ Control rats were given saline intragastrically at an equal volume. For CKD + Rb1 rats, ginsenoside Rb1 was administered intraperitoneally at a dose of 40 mg/kg/d, while an equal volume of saline was administered to CKD rats intraperitoneally. Bodyweights were assessed every 3 days, and the drug dose varied accordingly.

After 6 weeks, the animals were killed, and rat serum was collected to determine the blood urea nitrogen (BUN), creatinine (Cr), calcium, phosphorus and alkaline phosphatase (ALP) levels by an autoanalyzer (Chemray 240; Rayto Life and Analytical Sciences Co., Ltd). The abdominal arteries were excised for further analysis.

### 2.4 Alizarin red S staining and von Kossa staining

Vascular smooth muscle cell calcification was induced using 10 mmol/L β‐GP for 12 days as described above.^25^ After 12 days, VSMCs were washed with PBS three times and fixed in 70% ethanol for 60 minutes at room temperature. After rinsing with PBS, VSMCs were exposed to 1 mg/mL alizarin red S solution (pH 4.2) for another 60 minutes in the dark.

For artery calcification staining, 3 μm paraffin‐embedded artery sections were stained with alizarin red S solution for 10 minutes after the standard dewaxing procedure. Von Kossa staining of artery sections was also performed to confirm calcification. Dewaxed sections were exposed to a 5% silver nitrate solution and were placed under an ultraviolet light for 2 hours.

### 2.5 ALP activity and calcium content detection

The Alkaline Phosphatase Assay Kit (A059‐2) and Calcium Assay Kit (C004‐2) were from Nanjing Jiancheng Bioengineering Institute. The ALP activity and calcium content were determined according to the manufacturer's instructions. The results were then normalized by the protein content determined by the BCA Protein Assay Kit (PC0020) from Solarbio.

### 2.6 Western blot analysis

Vascular smooth muscle cells or rat arteries were lysed for 30 minutes on ice in lysis buffer containing 50 mmol/L Tris (pH 7.4), 150 mmol/L NaCl, 1% Triton X‐100, 1% sodium deoxycholate, 0.1% SDS, 2 mmol/L sodium pyrophosphate, 25 mmol/L β‐glycerophosphate, 1 mmol/L EDTA, 1 mmol/L Na_3_VO_4_, 0.5 μg/mL leupeptin and 0.1 mmol/L PMSF (R0010) from Solarbio. The supernatant was collected after centrifugation at 12 000 *g* for 10 minutes at 4°C.

Proteins were separated by 10% sodium dodecyl sulphate‐polyacrylamide gel electrophoresis (SDS‐PAGE) and then transferred to polyvinylidene difluoride (PVDF) membranes from Millipore. After blocking in 5% nonfat milk for 1 hour at room temperature, the PVDF membranes were probed with primary antibodies against α‐SMA (1:1000 dilution), calponin 1 (1:1000 dilution), RUNX2 (1:500 dilution), β‐catenin (1:500 dilution), phospho‐β‐catenin (Ser675) (1:1000 dilution), GSK‐3β (1:1000 dilution), phospho‐GSK‐3β (Ser9) (1:1000 dilution), PPAR‐γ (1:1000 dilution), histone‐H3 (1:1000 dilution) and GAPDH (1:1000 dilution) overnight. The membranes were then washed with TBS‐T, followed by an incubation with a horseradish peroxidase‐conjugated secondary antibody (1:8000 dilution) (ZSGB‐BIO) for 1.5 hours at room temperature; then, the membranes were developed with chemiluminescence and were stripped and reprobed when necessary.

### 2.7 Immunohistochemistry (IHC)

Following the standard procedure, paraffin‐embedded rat artery sections were rehydrated by dimethylbenzene and gradient ethanol. Then, 0.05 mol/L sodium citrate buffer (pH 6.0) was introduced for heat‐mediated antigen retrieval. Slides were submerged in 3% hydrogen peroxide for 10 minutes to remove endogenous peroxidase. After a wash step, the slides were blocked with 10% goat serum (ZLI‐9021; ZSGB‐BIO) for 30 minutes at 37°C, followed by an overnight incubation with primary antibodies against α‐SMA (1:500 dilution), calponin 1 (1:200 dilution) and RUNX2 (1:100 dilution) at 4°C in a humid box. After 30 minutes of incubation with the appropriate secondary antibody at 37°C, the slides were reacted with DAB solution (ZSGB‐BIO). Haematoxylin was applied to counterstain the nucleus. The tissue sections were visualized under a Nikon Eclipse 80i microscope equipped with a digital camera (DS‐Ri1; Nikon) and were analysed with Image‐Pro Plus 6.0 software.

### 2.8 Immunofluorescence (IF) and confocal microscopy

After rehydration, heat antigen retrieval, and 3% H_2_O_2_ treatment, the artery sections were permeabilized with 0.3% Triton X‐100 (T8200; Solarbio) for 15 minutes. After washing with PBS, the slides were then blocked and probed with the appropriate antibodies as described in the IHC procedure. Antibodies against β‐catenin (1:100 dilution) and PPAR‐γ (1:100 dilution) were used in this study. After washing, the slides were incubated with a secondary antibody (1:200 dilution, Proteintech Group) for 1 hour at 37°C. The slides were covered by a drop of Fluoroshield Mounting Medium containing 40,6‐diamidino‐2‐phenylindole (DAPI; Abcam) before being observed with laser scanning confocal microscopy (LSM710; Zeiss).

For the VSMC IF procedure, cells were seeded onto coverslips in a 24‐well plate and treated as described above. After fixation with immunostaining fixation solution (P0098; Beyotime Biotechnology) for 1 hour at room temperature, VSMCs were blocked, probed with antibodies, stained with DAPI and observed as artery sections.

### 2.9 Statistical analysis

All experiments were independently repeated at least three times. Data are expressed as the mean ± SEM. GraphPad Prism 6.0 was used to analyse the data and draw figures. Multiple group data were analysed by one‐way ANOVA, followed by Tukey's post hoc test. *P* < .05 was considered significantly different.

## 3 RESULTS

### 3.1 Rb1 reduces calcium deposition in vivo and in vitro

Rat CKD was induced by adenine gavage. As shown in Table 1, adenine‐treated rats developed severe CKD, with significantly higher BUN and creatinine levels (*P* < .01) than the control rats. Moreover, serum calcium levels in CKD rats did not change, whereas the serum phosphorus (*P* < .01) and ALP (*P* < .05) levels were increased. Rb1 administration did not ameliorate CKD rats' renal function but lowered the serum phosphorus levels (*P* < .05).

**Table 1:** Serum biochemical parameters

	Control	CKD	CKD + Rb1
BUN (mg/dL)	18.7 ± 1.041	28.49 ± 1.488**	31.07 ± 1.987**
Cr (μmol/L)	72.97 ± 2.247	86.83 ± 2.913**	83.16 ± 0.981*
Calcium (mmol/L)	2.498 ± 0.007	2.495 ± 0.008	2.467 ± 0.021
Phosphorus (mmol/L)	2.627 ± 0.035	2.907 ± 0.061**	2.682 ± 0.058#
ALP (U/L)	215.9 ± 19.01	290.3 ± 6.631*	287.5 ± 24.88*

Note

Rat serum levels of blood urea nitrogen (BUN), creatinine (Cr), calcium, phosphorus and alkaline phosphatase (ALP) were measured by an autoanalyzer. Values are means ± SEMs (n = 5).

* /^**^ *P* < .05/.01 vs Control group.

^#^ *P* < .05 vs CKD group.

**Figure 1:**
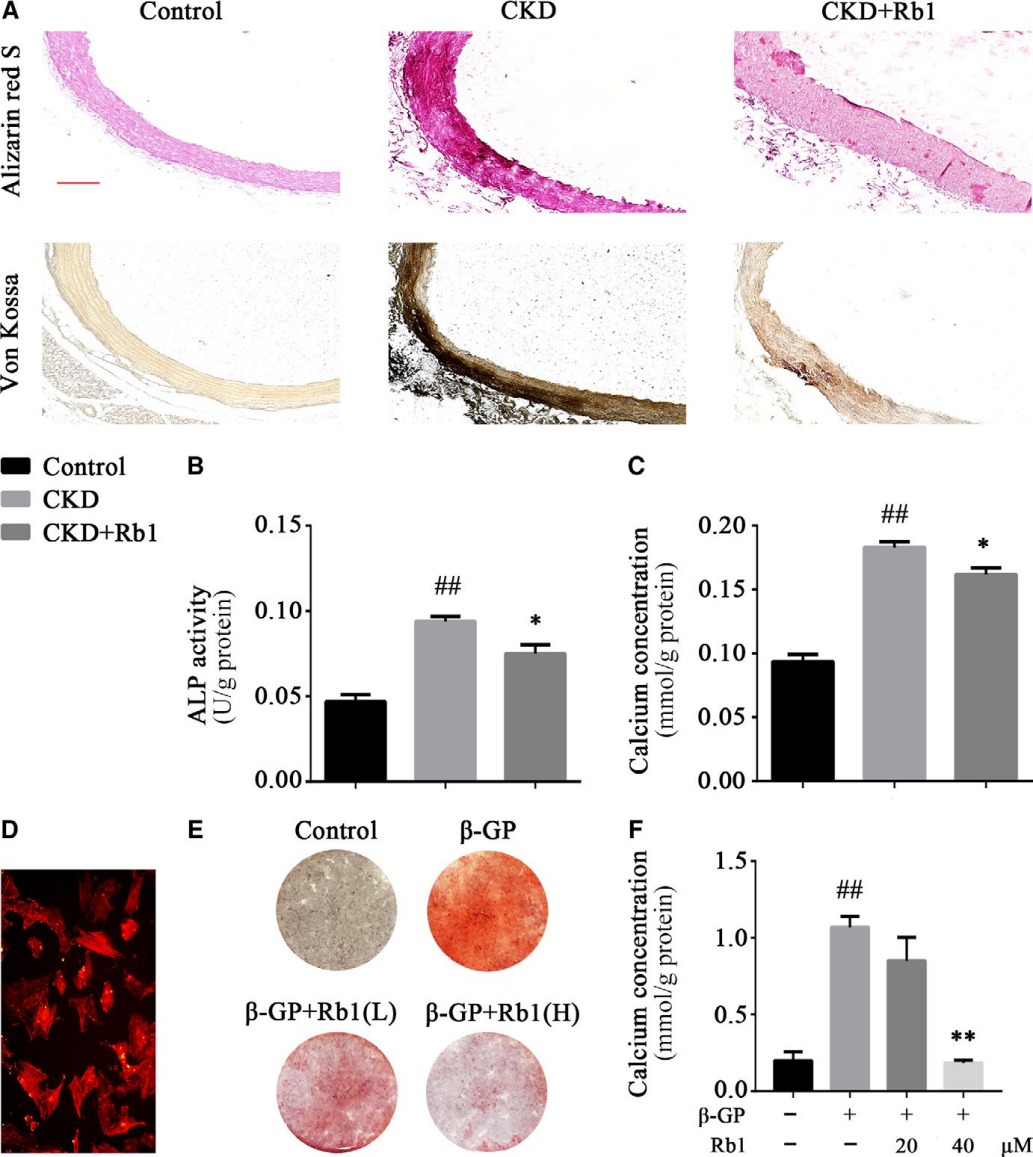
Rb1 reduces calcium deposition in vivo and in vitro. A, Alizarin red S staining and von Kossa staining of rat abdominal aortas (scale bar: 50 μm). B, Quantitative analysis of ALP activity in rat aortas normalized to the protein content. C, Quantitative analysis of calcium concentrations in rat aortas normalized to the protein content. D, Primary rat VSMCs immunostained for α‐SMA. E, Alizarin red S staining of VSMCs after β‐GP induction for 12 days. F, Quantitative analysis of calcium concentrations of VSMCs after β‐GP induction for 6 d, normalized to the protein content. Data are the mean ± SEM. ^#^ *P* < .05, ^##^ *P* < .01 vs control; *P < .05, **P < .01 vs β‐GP. CKD indicates chronic kidney disease; β‐GP, β‐glycerophosphate; Rb1(L), low dose (20 μmol/L) of Rb1; Rb1(H), high dose (40 μmol/L) of Rb1

To assess vascular medial calcification in adenine‐induced CKD rats, alizarin red S staining and von Kossa staining were performed. As shown in Figure 1A, adenine‐induced CKD rats developed more severe medial calcification than the control, while Rb1 intervention reduced this pathological change. Furthermore, ALP activity (Figure 1B) and calcium content (Figure 1C) assays of rat artery homogenates also demonstrated that Rb1 administration alleviated the degree of calcification in vivo (*P* < .05).

Primary VSMCs were extracted for in vitro experiments. After positive immunostaining for α‐SMA (Figure 1D), VSMCs were stimulated with 10 mmol/L β‐GP with different concentrations of Rb1 (20 and 40 μmol/L). Consistent with our findings in rats, Rb1 reduced the calcium deposition of VSMCs, as proven by alizarin red S staining (Figure 1E) and the calcium content assay (Figure 1F; *P* < .01). Notably, Rb1 (40 μmol/L) treatment remarkedly reduced VSMC calcification compared to Rb1 (20 μmol/L) treatment.

Taken together, the results in this section suggest that ginsenoside Rb1 reduces the calcium deposition of CKD‐associated VC both in vivo and in vitro.

### 3.2 Rb1 inhibits VSMC phenotype switching in vivo and in vitro

**Figure 2:**
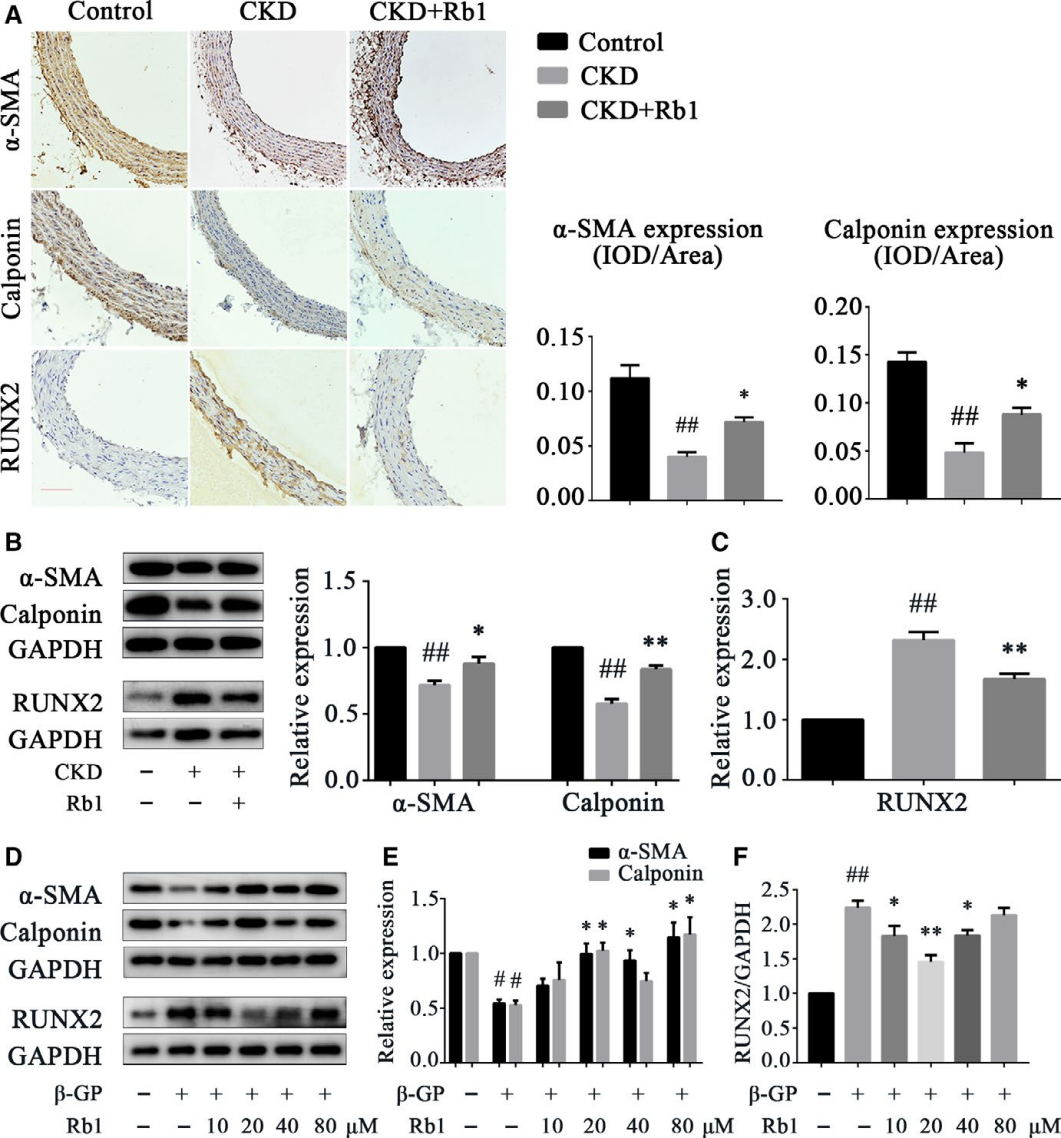
Rb1 inhibits VSMC phenotype switching in vivo and in vitro. A, Representative immunohistochemical staining of α‐SMA, calponin and RUNX2 in rat aortas; semiquantitative analysis of α‐SMA and calponin (n = 5 per group; scale bar: 100 μm). B and C, Representative Western blot bands and semiquantitative analysis of α‐SMA, calponin and RUNX2 in rat aortas (n = 5 per group). D‐F, Representative Western blot bands and semiquantitative analysis of α‐SMA, calponin and RUNX2 in VSMCs after β‐GP induction for 72 h. Data are presented as the mean ± SEM. ^#^ *P* < .05, ^##^ *P* < .01 vs control; *P < .05, **P < .01 vs CKD/β‐GP. CKD indicates chronic kidney disease; RUNX2, runt‐related transcription factor 2; β‐GP, β‐glycerophosphate; GAPDH, glyceraldehyde‐3‐phosphate dehydrogenase

Calcification has been demonstrated to be a multifactorial vascular change rather than a simple calcium deposition, and the osteogenic transformation of VSMC is one of the key mechanisms involved in VC.^3^ Here, to investigate the role of ginsenoside Rb1 in VSMC phenotype switching, we implemented experiments both in vivo and in vitro. A‐smooth muscle actin (α‐SMA) and calponin were detected as classical VSMC contractive markers, while runt‐related transcription factor 2 (RUNX2) was detected as an induced osteogenic marker. From the IHC results (Figure 2A), it can be observed that the diminished VSMC contractive markers in CKD rat arteries were rescued in the arteries of CKD + Rb1 rats (*P* < .05). Additionally, the induced expression of RUNX2 in CKD rats was reduced in CKD + Rb1 rats. Western blots further verified these results (Figure 2B,C), indicating that Rb1 inhibits VSMC osteogenic transformation in the process of VC. In addition, in vitro experiments also showed that in β‐GP‐induced VSMC calcification, Rb1 both up‐regulated the expression of contractive markers and down‐regulated the expression of osteogenic markers in a dose‐dependent manner (Figure 2D‐F). Interestingly, a low dose of Rb1 (20 μmol/L) was proved to be more effective in inhibiting VSMC phenotype switching according to the statistical analysis.

These results suggest that ginsenoside Rb1 inhibits the osteogenic transformation of VSMCs in vivo and in vitro.

### 3.3 The Wnt/β‐catenin pathway is activated in β‐GP‐induced VSMC calcification

**Figure 3:**
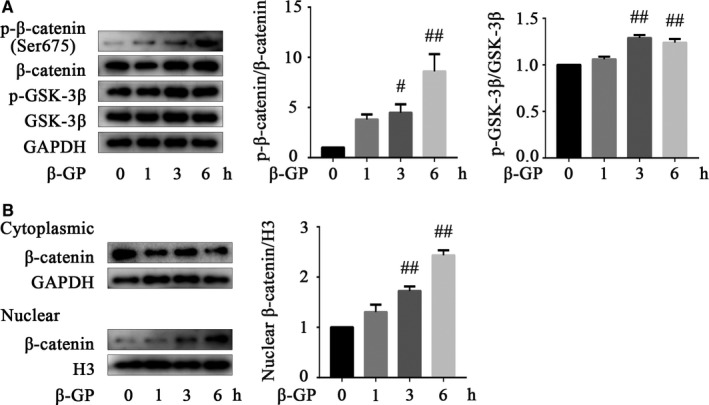
The Wnt/β‐catenin pathway is activated in β‐GP‐induced VSMC calcification. A, Representative Western blot bands of p‐GSK‐3β (Ser9), GSK‐3β, p‐β‐catenin (Ser675) and β‐catenin in VSMCs after β‐GP induction for different times. B, Representative Western blot bands of cytoplasmic and nuclear β‐catenin in VSMCs after β‐GP induction for different times. Data are presented as the mean ± SEM. ^#^ *P* < .05, ^##^ *P* < .01 vs control. P‐GSK‐3β indicates phosphorylated glycogen synthase kinase‐3β; β‐GP, β‐glycerophosphate; H3, histone‐H3; GAPDH, glyceraldehyde‐3‐phosphate dehydrogenase

Sufficient studies have shown that the CKD procalcific environment induces Wnt/β‐catenin pathway activation, which in turn, promotes the development of VC.^8^, ^9^, ^10^, ^26^ Here, we demonstrated that β‐GP stimulation for 6 hours promoted the phosphorylation of GSK‐3β (Ser9) and β‐catenin (Ser675) (*P* < .01; Figure 3A). Ser675 phosphorylation of β‐catenin by protein kinase A (PKA) promotes the transcriptional activity (TCF/LEF transactivation) of β‐catenin.^27^, ^28^ Therefore, these results confirmed the activation of the Wnt/β‐catenin pathway in VSMC calcification. Furthermore, Western blots revealed that the expression of nuclear β‐catenin increased in a time‐dependent manner, while there was no significant change observed in the expression of cytoplasmic β‐catenin, indicating that nuclear translocation was enhanced (*P* < .01; Figure 3B). Taken together, these results indicate that the Wnt/β‐catenin pathway was activated during β‐GP‐induced VSMC calcification.

### 3.4 Rb1 activates PPAR‐γ and down‐regulates the Wnt/β‐catenin pathway

**Figure 4:**
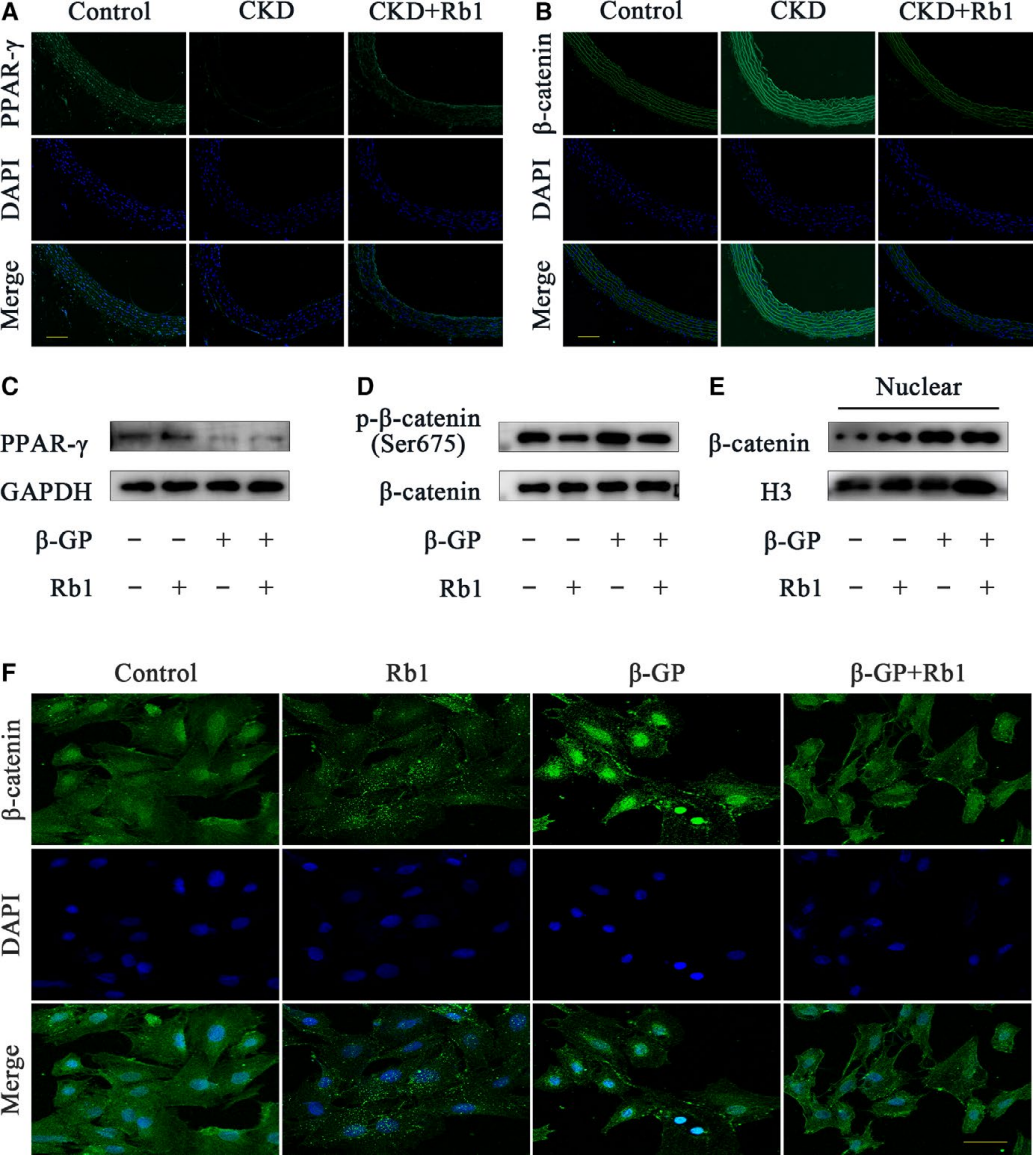
Rb1 activates PPAR‐γ and down‐regulates the Wnt/β‐catenin pathway. A and B, Representative immunofluorescence staining of PPAR‐γ and β‐catenin in rat aortas (scale bar: 100 μm). C‐E, Representative Western blot bands of PPAR‐γ (C), p‐β‐catenin/ β‐catenin (D), nuclear β‐catenin (E) with or without 40 μmol/L Rb1 in the presence or absence of β‐GP for 6 h. F, Confocal microscopy of the immunofluorescence staining of β‐catenin in VSMCs with or without 40 μmol/L Rb1 in the presence or absence of β‐GP for 6 h (scale bar: 100 μm). CKD indicates chronic kidney disease; DAPI, 40,6‐diamidino‐2‐phenylindole; β‐GP, β‐glycerophosphate; H3, histone‐H3; GAPDH, glyceraldehyde‐3‐phosphate dehydrogenase

Based on our existing findings, we next aimed to reveal the intracellular mechanism mediating the VC‐protective effect of Rb1. Therefore, we detected the expression of the Wnt/β‐catenin pathway and the nuclear receptor PPAR‐γ. As presented in Figure 4, IF showed that the decreased expression of PPAR‐γ in CKD rats (vs control) was increased in the CKD + Rb1 group, while compared with that in the CKD group, β‐catenin expression was decreased in CKD + Rb1 rats (Figure 4A,B). Experiments in VSMCs further validated these results. The expression of PPAR‐γ was enhanced by Rb1 stimulation for 6 hours (*P* < .05; Figures 4C and S1A), whereas Ser675 phosphorylation of β‐catenin was inhibited by Rb1 in the presence (*P* < .05) or absence (*P* < .01) of β‐GP (Figures 4D and S1B). Notably, the nuclear translocation of β‐catenin was also blocked by Rb1 as illustrated by Western blots (*P* < .05; Figures 4E and S1C) and confocal microscopy (Figure 4F). In brief, these data indicate that Rb1 down‐regulates the Wnt/β‐catenin pathway and activates PPAR‐γ.

### 3.5 Rb1 ameliorates VC by down‐regulating the Wnt/β‐catenin pathway

**Figure 5:**
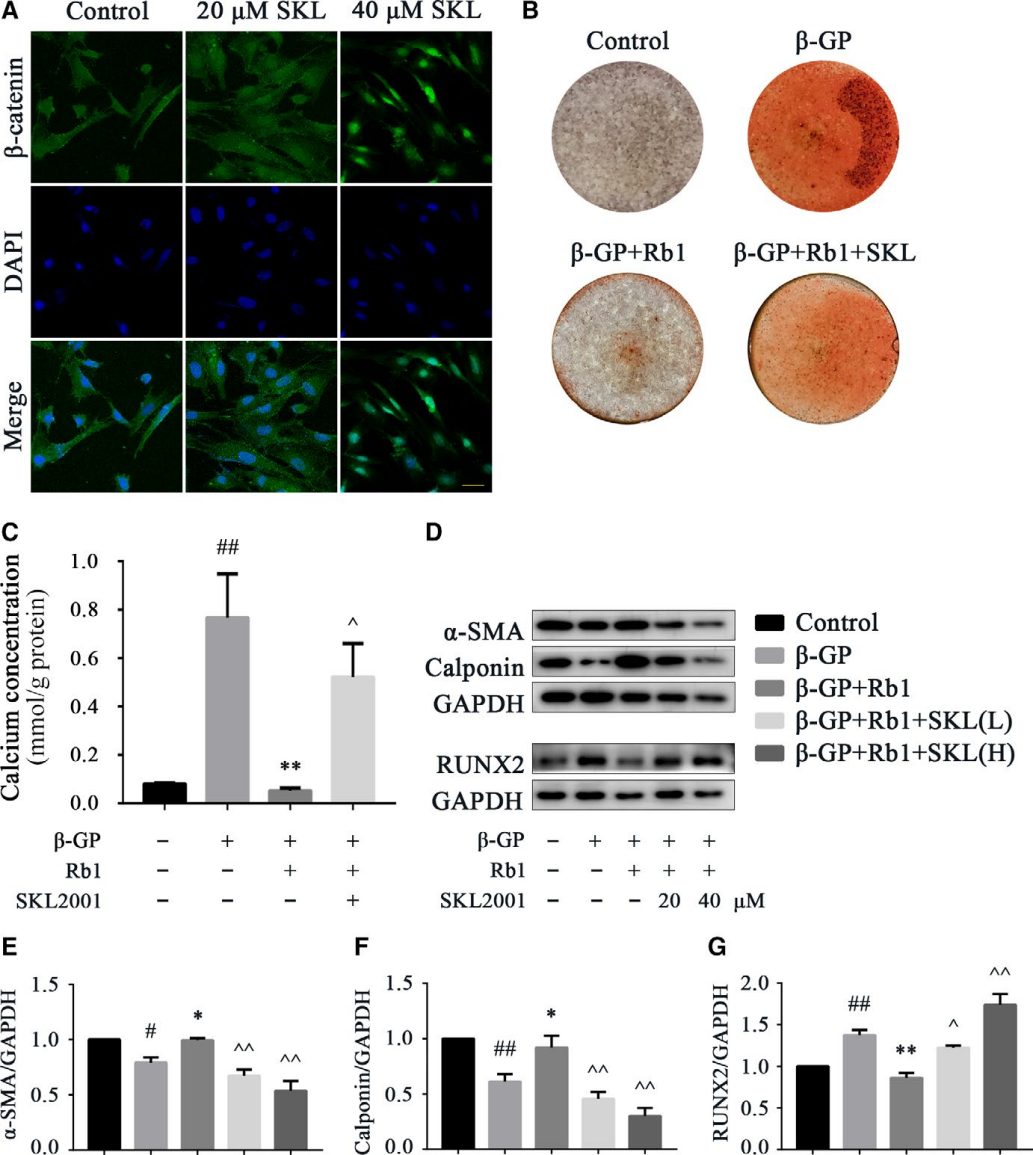
Rb1 ameliorates VC by down‐regulating the Wnt/β‐catenin pathway. A, Representative immunofluorescence staining of β‐catenin in VSMCs with different concentrations of SKL2001 for 6 h (scale bar: 100 μm). B, Alizarin red S staining of VSMCs after induction with different stimuli for 12 days: 40 μmol/L Rb1 and 40 μmol/L SKL2001. C, Quantitative analysis of the calcium concentrations of VSMCs after induction with different stimuli for 6 d, normalized to the protein content, and 40 μmol/L Rb1 and 40 μmol/L SKL2001 were used. D‐G, Representative Western blot bands and semiquantitative analysis of α‐SMA, calponin and RUNX2 in VSMCs after induction with different stimulation for 72 h. Data are presented as the mean ± SEM. n = 3‐5, ^#^ *P* < .05, ^##^ *P* < .01 vs control; *P < .05, **P < .01 vs β‐GP; ^*P* < .05, ^^*P* < .01 vs β‐GP + Rb1. SKL indicates SKL2001; DAPI, 40,6‐diamidino‐2‐phenylindole; β‐GP, β‐glycerophosphate; RUNX2, runt‐related transcription factor 2; GAPDH, glyceraldehyde‐3‐phosphate dehydrogenase; SKL(L), low dose (20 μmol/L) of SKL2001; SKL(H), high dose (40 μmol/L) of SKL2001

Having obtained evidence of the anticalcification effect of Rb1 and the potential involved pathway, we next explored whether pathway alteration changed the VC‐protective effect of Rb1. As presented, a 6‐hour stimulation with SKL2001 (20 and 40 μmol/L), a Wnt/β‐catenin pathway agonist, was observed to promote the nuclear translocation of β‐catenin in VSMCs (Figure 5A). Interestingly, after adding SKL2001, calcium deposition was aggravated, as proven by alizarin red S staining (Figure 5B) and a calcium content assay (Figure 5C), and VSMC phenotype switching was remarkably exacerbated (Figure 5D‐G). VSMC contractive markers, α‐SMA and calponin, were reduced in β‐GP‐induced calcification, and Rb1 (40 μmol/L) treatment alleviated this reduction in α‐SMA and calponin, which were then eliminated by SKL2001. Consistently, the calcification‐induced enhancement of the osteogenic marker RUNX2 was relieved by 40 μmol/L Rb1 and then increased after SKL2001 addition. In summary, Rb1 ameliorated VC through a Wnt/β‐catenin‐dependent pathway.

### 3.6 Rb1 inhibits the Wnt/β‐catenin pathway through the activation of nuclear receptor PPAR‐γ

**Figure 6:**
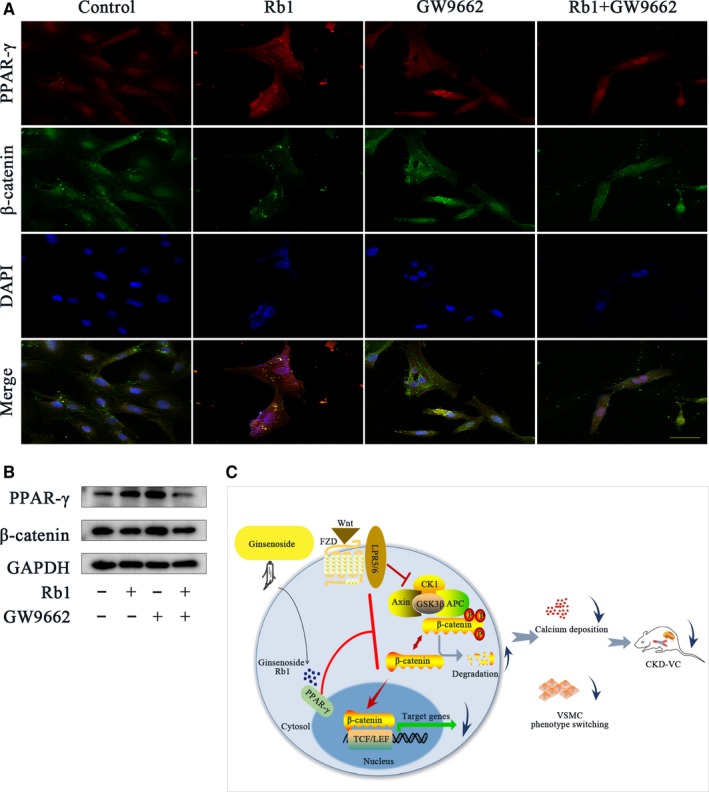
Rb1 inhibits the Wnt/β‐catenin pathway through the activation of nuclear receptor PPAR‐γ. A, Confocal microscopy of the immunofluorescence staining of PPAR‐γ (red) and β‐catenin (green) with different treatments for 24 h: 5 μmol/L GW9662 and 40 μmol/L Rb1 (scale bar: 100 μm). B, Representative Western blot bands of PPAR‐γ and β‐catenin in VSMCs treated for 24 h: 5 μmol/L GW9662 and 40 μmol/L Rb1. C, Schematic diagram of the effect of Rb1 on VSMC calcification. DAPI indicates 40,6‐diamidino‐2‐phenylindole; GAPDH, glyceraldehyde‐3‐phosphate dehydrogenase

The crosstalking between PPAR‐γ and the Wnt signalling pathway has been widely discussed in various diseases.^29^, ^30^, ^31^ Here, IF double staining and Western blot analyses were performed to illustrate the interaction between PPAR‐γ and β‐catenin. As shown in the IF results, PPAR‐γ expression was increased with Rb1 (40 μmol/L) treatment for 24 hours, while costimulation of 5 μmol/L GW9662, a selective PPAR‐γ antagonist, blocked this effect. Consistently, the reduced expression of β‐catenin by Rb1 administration was augmented with GW9662 costimulation (Figure 6A). Moreover, Western blotting for PPAR‐γ and β‐catenin further confirmed that PPAR‐γ activation mediated the Rb1 inhibition of β‐catenin (*P* < .05; Figures 6B and S1D). Interestingly, GW9662 treatment alone had little impact on PPAR‐γ (Figure 6A) and even increased PPAR‐γ expression (Figure 6B). A possible explanation for this might be that GW9662 acts via inhibiting ligand binding to PPAR‐γ, while in the absence of these ligands, the expression of PPAR‐γ increased due to feedback.

Taken together, Rb1 inhibits the Wnt/β‐catenin pathway by activating the nuclear receptor PPAR‐γ.

## 4 DISCUSSION

In the present study, we demonstrated that ginsenoside Rb1 ameliorated calcium deposition and VSMC phenotype switching in both adenine‐induced CKD rats and in rat VSMCs. Rb1 exerted an anticalcific effect through inhibition of the Wnt/β‐catenin pathway via activating PPAR‐γ, which provides new insights into the potential pharmacological interventions of VC.

Vascular calcification poses a great threat to human health due to its high morbidity and mortality.^32^, ^33^ Studies have shown that CKD patients are prone to VC even in the early stage (25% in stage 3 and 35% in stage 4), and the prevalence rises rapidly once CKD patients start dialysis (over 50%).^34^, ^35^ Consequently, the increased arterial stiffness inflicts left ventricular hypertrophy (LVH) and augmented pulse pressure, which affects coronary perfusion and escalates stroke risk. Calcium deposition in tunica media is the most prominent histological feature of medial VC. Ginsenosides have been widely used as tonic botanical products in the West. PPD and PPT are two subtypes of ginsenosides in terms of chemical structure.^36^ Rb1, a PPD type of ginsenoside, is known for its beneficial properties in the CNS, cardiovascular and endocrine systems.^37^, ^38^, ^39^ Recently, Rb1 has been reported to protect arterial function in pulmonary hypertension and atherosclerosis models.^16^, ^17^ In addition, Xu et al^18^ reported that Rb1 alleviated early CKD progression by modulating oxidative stress and inflammation. However, the effect of Rb1 on CKD‐VC has not been elucidated. In this study, we demonstrated that Rb1 reduced calcium deposition as well as ALP activity and calcium concentration in CKD rat arteries. Further, the results in primary rat VSMCs also confirmed the protective role of Rb1 in calcium deposition. All of the above findings strengthen the knowledge of the protective property of ginsenoside Rb1 in vascular diseases.

Sufficient evidence has shown that VC involves a pluriform range of pathobiological processes rather than simple calcium deposition, among which, VSMC switching into osteoblast‐like cells plays a critical role. In the present study, we showed that the contractile VSMC markers α‐SMA and calponin were reduced in CKD rat aortas and calcified VSMCs compared to those in the control rats, and we showed that Rb1 increased the expression of α‐SMA and calponin in a dose‐dependent manner. RUNX2, an essential transcription factor in osteoblast differentiation and skeletal morphogenesis,^40^ was detected here as an osteogenic marker. We proved that the up‐regulated expression of RUNX2 was suppressed by Rb1 both in vivo and in vitro, indicating that Rb1 inhibited VSMC phenotype switching of VC. Interestingly, it was demonstrated that ginsenosides promote osteogenic differentiation.^41^, ^42^, ^43^, ^44^ Ginsenoside Rg1 promotes rat bone marrow mesenchymal stem cell (rBMSC) osteogenic differentiation by activating the GR/BMP‐2 signalling pathway.^41^ Moreover, ginsenosides Rg3, Rd and Rh2(S) were reported to induce the differentiation and mineralization of MC3T3‐E1 cells.^42^, ^43^, ^44^ Nevertheless, none of the studies above investigated the effect of Rb1 on VSMC phenotype switching or the role of the PPAR‐γ/β‐catenin pathway in VC.

To further explore the underlying mechanism of the anticalcific effect of Rb1, we next focused on β‐catenin/PPAR‐γ crosstalk. Signal transduction via the canonical Wnt pathway includes cytoplasmic stabilization and the nuclear translocation of β‐catenin. Interacting with the T cell factor/lymphoid enhancer factor (TCF/LEF) family, β‐catenin regulates the transcription of downstream genes, thereby governing cellular fate determination processes, such as osteogenic differentiation of MSCs.^4^ Growing evidence has shown that the Wnt/β‐catenin pathway plays a key role in VC.^8^, ^9^, ^10^ It has been reported that the Wnt/β‐catenin pathway mediates the induction of CKD‐VC by bone morphogenetic protein‐2 (BMP2).^9^ High phosphate was reported to activate WNT/β‐catenin signalling, which promoted VC via directly modulating Runx2 gene expression.^7^ Moreover, several studies have demonstrated that the inhibition of the WNT/β‐catenin pathway reduces VC.^10^, ^45^ In this study, the activated Wnt/β‐catenin pathway and enhanced nuclear translocation of β‐catenin were observed in β‐GP‐induced VSMC calcification, which is in accordance with the study by Cai et al.^7^

PPAR‐γ has been reported to regulate glucolipid metabolism and to determine the differentiation of various cell types.^46^, ^47^ Recently, studies have reported that PPAR‐γ is involved in vascular inflammation and atherosclerosis.^48^, ^49^ More importantly, activation of PPAR‐γ has been demonstrated to maintain the VSMC phenotype, and the down‐regulation of PPAR‐γ contributes to CKD‐associated VC.^8^, ^50^ Belonging to the steroidal saponin family, ginsenosides share a similar structure with steroid hormones, which can traverse the membrane and disrupt the genome.^36^ Furthermore, it has been reported that PPT may act as a PPAR‐γ ligand that is involved in the PPAR‐γ‐mediated transactivation of target genes.^51^ In the present study, we found that PPAR‐γ was significantly decreased in CKD rat aortas, whereas Rb1 up‐regulated PPAR‐γ gene expression. In contrast, the increased expression of β‐catenin was reversed by Rb1 treatment. In calcifying VSMCs, we found that Rb1 inhibited the activation of the Wnt/β‐catenin pathway as well as β‐catenin nuclear translocation, indicating that Rb1 activated PPAR‐γ and inhibited the Wnt/β‐catenin pathway. Furthermore, we also noticed that after stimulation with SKL2001, the anticalcific effect of Rb1 on both calcium deposition and VSMC phenotype switching was compromised, suggesting that Rb1 alleviated VC by inhibiting the Wnt/β‐catenin pathway. Studies have implied that ginsenosides function through nuclear receptors.^19^, ^52^, ^53^ Our previous study also suggests that ginsenoside Rb1 activates PPAR‐γ in HUVECs.^21^ Interestingly, new metabolites of Rb1 by microbial transformation have been reported to activate the Wnt/β‐catenin pathway, which mediates the induction of osteogenic differentiation in MC3T3‐E1 cells.^54^ A possible explanation for this discrepancy might be that the process of microbial transformation altered not only biochemical structures but also certain properties of Rb1, and the effects of these new derivatives on VSMC calcification doubtlessly need to be further determined.

The interplay between PPAR‐γ and the Wnt signalling pathway has been extensively discussed. For example, the PPAR‐γ and Wnt pathways orchestrate the adipogenesis and osteogenesis of MSCs.^12^ Jiajian Liu, *et al* reported that the functional interaction between β‐catenin and PPAR‐γ involved the TCF/LEF‐binding domain of β‐catenin and a catenin‐binding domain (CBD) within PPAR‐γ.^55^ In this study, we substantiate the interaction between PPAR‐γ and β‐catenin by GW9662 intervention, indicating that Rb1 inhibited the Wnt/β‐catenin pathway through the upstream activation of PPAR‐γ.

Nevertheless, insufficiency of this study remains in that, despite being a generally accepted model of CKD, adenine‐induced CKD rats suffered faster weight loss and less extensive VC than clinical CKD patients due to the gavage modelling time being relatively intense and limited, which requires future studies for further exploration.

Overall, as illustrated in the schematic diagram (Figure 6C), this study first demonstrated that ginsenoside Rb1 ameliorates CKD‐associated VC by inhibiting the Wnt/β‐catenin pathway by activating PPAR‐γ. These promising findings provide novel insights into the potential conversion of natural products into clinical therapeutics for VC.

## CONFLICT OF INTEREST

None.

## AUTHOR CONTRIBUTIONS

JXP and LHX conceived and designed the experiments. ZP, ZXY, GMQ, WL, ZZH and GR performed the experiments. ZXY, LZW and DM researched the data and contributed to the discussion. ZP and ZXY analysed the data. DHY contributed reagents, materials and analytic tools. ZP and LHX wrote the manuscript. JXP and LHX reviewed and edited the manuscript.
