# Supplementary figures and images for: Ginsenoside Rb1 ameliorates CKD‐associated vascular calcification by inhibiting the Wnt/β‐catenin pathway
Source: J Cell Mol Med. 2019 Aug 19;23(10):7088–98. doi: 10.1111/jcmm.14611 (PMC6787443; doi:10.1111/jcmm.14611)

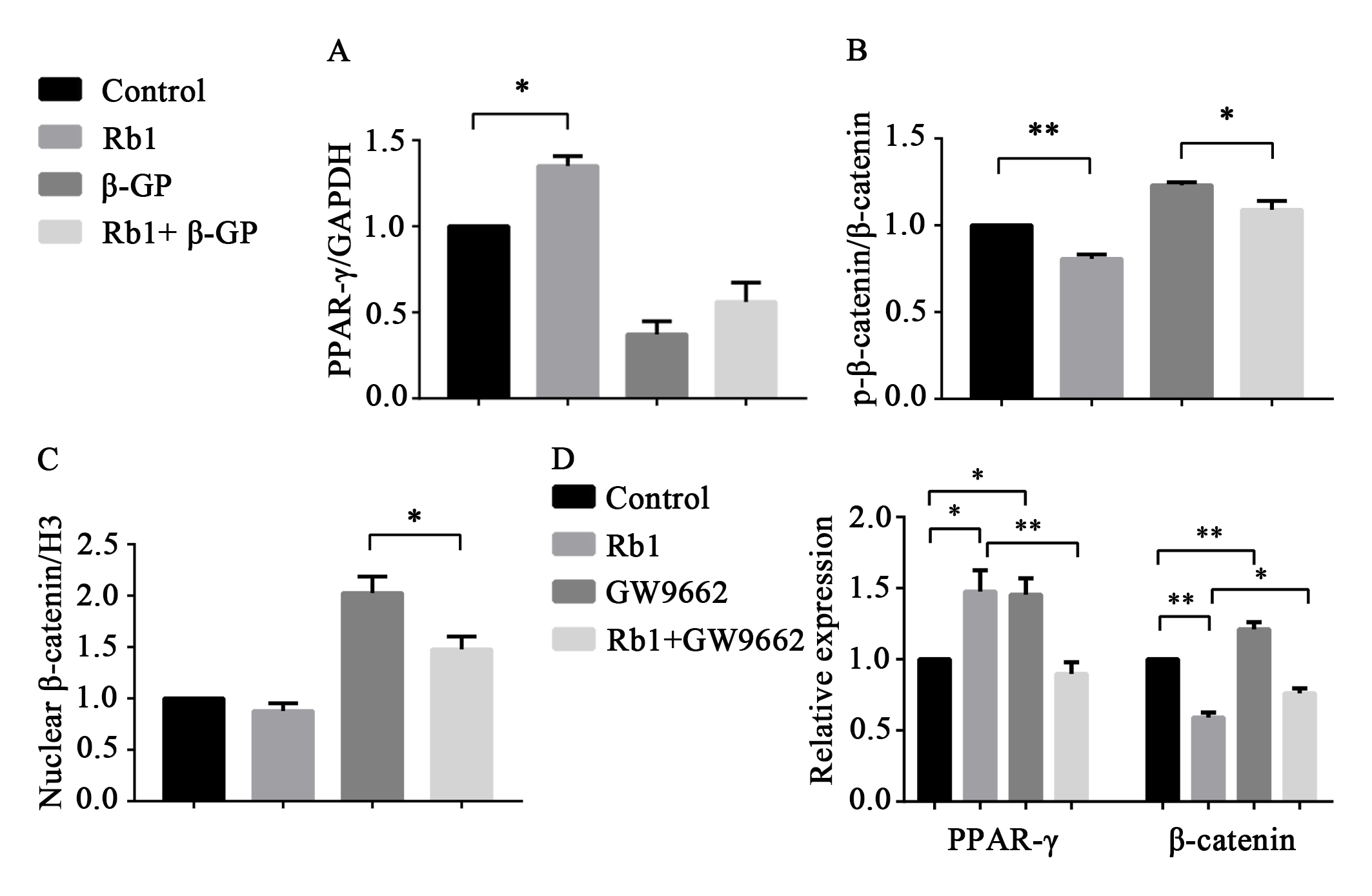

Supplement: Supplementary file 1 [file JCMM-23-7088-s001.tif]
